# Gene editing of the thioester reductase step in the biosynthesis of lysergic acid amides

**DOI:** 10.1371/journal.pone.0334651

**Published:** 2025-10-29

**Authors:** Lauren M. Bish, Jessica L. Fuss, Daniel G. Panaccione

**Affiliations:** West Virginia University, School of Natural Resources and the Environment, Morgantown, West Virginia, United States of America; Université de Montréal: Universite de Montreal, CANADA

## Abstract

Ergot alkaloids derived from lysergic acid are important in agriculture, as food and feed contaminants, and in medicine, as the foundation of several pharmaceuticals. The fungus *Metarhizium brunneum* makes several lysergic acid amides, with lysergic acid α-hydroxyethylamide (LAH) being produced in by far the highest concentration. The multifunctional enzyme lysergyl peptide synthetase 3 (Lps3) has multiple domains that play important roles in lysergic acid amide synthesis. We hypothesized a role for the reductase domain of Lps3 in liberating LAH from an enzyme-bound precursor and tested this hypothesis with CRISPR/Cas9-based gene editing experiments. We transformed *M. brunneum* with a Cas9/single guide RNA complex and a donor DNA that replaced the tyrosine at the active site of the reductase domain of Lps3 with a phenylalanine. Sanger sequencing of edited and wild-type genes demonstrated successful editing of the reductase domain without non-target mutations in Lps3. High performance liquid chromatography of the edited strain showed a significant reduction of LAH and accumulation of the precursor lysergic acid. The phenotype was similar when the edited allele of *lpsC* was in a wild-type background or in backgrounds with late pathway genes *easO* or *easP* knocked out, except no LAH was detectable when the edit was in the *easO* knockout background. The data demonstrate that the reductase domain plays a key role or roles in formation of LAH. The abundant lysergic acid accumulating in the mutants, as opposed to later pathway intermediates in LAH biosynthesis (such as lysergyl-alanine), indicated severe debilitation of Lps3. The data indicate a requirement for the reductase domain of Lps3 in synthesis of lysergic acid amides and demonstrate the feasibility of the CRISPR/Cas9-based approach for editing genes in *Metarhizium* species.

## Introduction

Ergot alkaloids are important agriculturally as toxic contaminants in grain and forage [1,2] and pharmaceutically for treating dementia, migraines, and several other disorders [3,4]. A diverse array of ergot alkaloids is produced as a result of the multiple branches of the ergot alkaloid pathway [1,3,4]. The branch that produces the products most infamous for contaminating grains and forage and for their role in licit and illicit pharmaceuticals is the branch derived from lysergic acid. Lysergic acid-derived ergot alkaloids can be classified into two groups: ergopeptines, in which lysergic acid is attached to a cyclic tripeptide of three amino acids that varies between, and defines, the members of the ergopeptine group; and, lysergic acid amides where lysergic acid is derivatized as an amide to alanine or a derivative thereof. Notable lysergic acid amides are ergonovine (called ergometrine in Europe), lysergic acid α-hydroxyethylamide (LAH), and ergine, which forms from hydrolysis of LAH (and perhaps to a lesser extent from hydrolysis of other lysergic acid derivatives) [4–6].

LAH is the main ergot alkaloid produced by *Metarhizium* species [6], *Claviceps paspali* [7,8], several *Periglandula* species [9–11], and a few recently characterized species of *Aspergillus* [12]. Most of the LAH-producing fungi also produce lesser quantities of ergonovine. In a sample of fungi that have the capacity to produce both LAH and ergonovine, a mean of over 90% of the lysergic acid amide accumulating was LAH [4]. *Claviceps purpurea* differs from most other lysergic acid amide-producing fungi in accumulating ergonovine but not LAH [1,13]. The most detailed biochemical analyses of lysergic acid amide biosynthesis have been done with *C. purpurea* and, thus, have focused on ergonovine biosynthesis as opposed to LAH [13].

**Fig 1:**
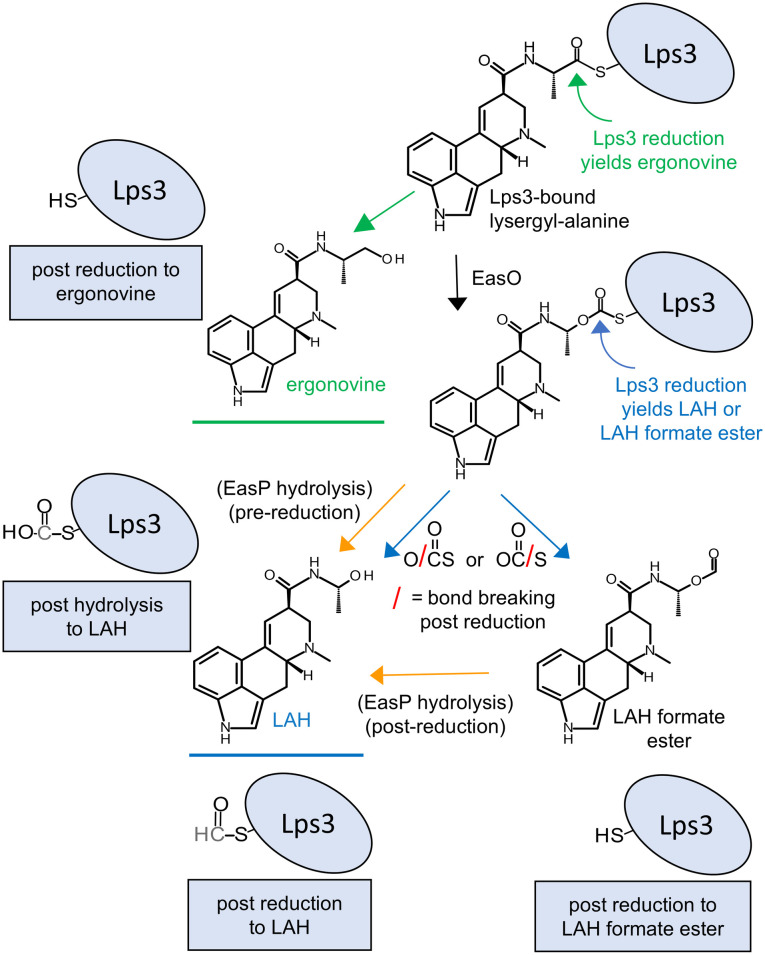
Model for derivation of lysergic acid amides from Lps3-bound lysergyl-alanine. Enzymes demonstrated (or hypothesized, in the case of EasP indicated in parentheses) to have roles are listed at relevant steps. Status of the terminus of the 4’-phosphopantetheine cofactor of Lps3 after different mechanisms of substrate removal is shown near relevant products. LAH, lysergic acid α-hydroxyethylamide.

Lysergic acid amides produced by fungi in the Clavicipitaceae are synthesized on a two-part peptide synthetase [13,14]. Lysergyl peptide synthetase 2 (encoded by *lpsB*) activates lysergic acid by adenylation and binds it as a thioester to an enzyme-bound 4’-phosphopantetheine cofactor. Lysergyl peptide synthetase 3 (Lps3; encoded by *lpsC*) binds L-alanine as a thioester and, in cooperation with Lps2, condenses lysergic acid with alanine to create an enzyme-bound lysergyl-alanine intermediate (Fig 1). In LAH producers, the Lps3-bound lysergyl-alanine then serves as a substrate for two competing pathway termination steps [4,15]. The first of the two termination mechanisms is most readily observed in organisms that lack the Bayer-Villiger monooxygenase encoded by *easO* [15]. Ortel and Keller [13] showed that in *C. purpurea* (which lacks the gene *easO*) the reductase domain of Lps3 reduces the carbonyl group of enzyme-bound lysergyl-alanine to an alcohol, resulting in formation and liberation of ergonovine and a fully reduced sulfhydryl group on the 4’-phosphopantetheine cofactor that is thus ready for another round of catalysis (Fig 1). Analyses of the Lps complex [13] as well as studies of reductase domains of other peptide synthetases [16–18] show that the two hydrogen molecules for each of the reductions are derived from components from two origins: a hydride ion (H-) from NADPH and a proton donated by a tyrosine at the active site of the reductase domain. The active site of the reductase domain involves a threonine-tyrosine-lysine catalytic triad similar to those observed in short chain dehydrogenase/reductases (SDR family of enzymes).

The second of the two termination mechanisms is proposed to occur in organisms that contain EasO, which modifies the Lps3-bound lysergyl-alanine substrate prior to it being acted on by the reductase domain [4,15]. In this proposed termination mechanism, LAH arises via a reduction of the same carbon reduced in ergonovine biosynthesis but after insertion of an oxygen into the alanine moiety by the Bayer-Villiger monooxygenase encoded by *easO* (Fig 1). Support of this hypothesis is provided by the elimination of LAH and a significant increase in ergonovine in strains of *M. brunneum* in which *easO* was knocked out by a CRISPR/Cas9 approach [15]. Moreover, labeling studies with deuterated alanine yielded products consistent with the activity of a Bayer-Villiger monooxygenase.

A third enzyme, an alpha/beta hydrolase fold protein encoded by the gene *easP*, contributes to LAH biosynthesis, though it is not required. Knockout of *easP* reduces LAH accumulation by approximately 60% [19]. Two potential mechanisms by which EasP could contribute to LAH accumulation without being essential have been proposed [4,19]: 1) It may hydrolyze LAH from the Lps3-bound product of EasO; and, 2) It may hydrolyze LAH from a hypothetical LAH formate ester that could form as an alternate product of reduction of the Lps3-bound intermediate by the reductase domain of Lps3 (Fig 1).

To test the role of the reductase domain of Lps3 in LAH biosynthesis while leaving the remainder of this multifunctional peptide synthetase intact, we mutated the active site tyrosine to phenylalanine by a CRISPR/Cas9-based gene editing approach and observed the effects of this mutation on ergot alkaloid accumulation. We also knocked out *easO* or *easP* in the Lps3 reductase domain-edited mutant to further investigate the roles of these late pathway enzymes in formation of LAH. Results of these editing experiments demonstrate a critical need for the reductase domain of Lps3 in LAH biosynthesis.

## Materials and methods

### Genetic modification and strain development

**Fig 2:**
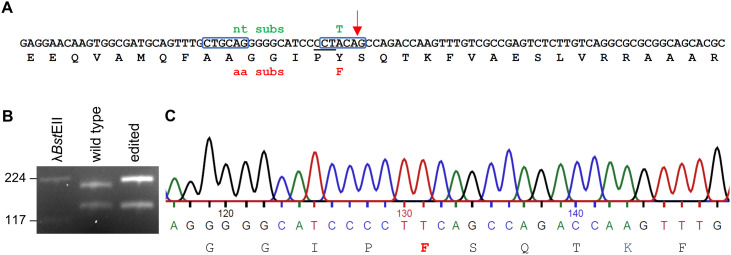
Editing of the active site of the reductase domain of Lps3. **(A)** Original and edited (with nt substitution indicated above in green and amino acid substitution below in red) versions of a portion of the reductase domain-encoding region of *lpsC*. PAM site (in reverse complement) indicated by the underlined CCT. The nucleotide sequence shown corresponds to the region used as the donor DNA, except the donor DNA contained the T indicated in green above the original A residue. The Cas9 cut site is shown with a red arrow. Two *Sfc*I sites are boxed; two additional relevant *Sfc*I sites flank the indicated sites and are not contained in this figure. **(B)** A 355-bp fragment containing the targeted region was amplified from wild-type and edited loci, and amplicons were treated with the restriction enzyme *Sfc*I. Editing of the active site tyrosine to a phenylalanine eliminated an *Sfc*I recognition site (C^TRYAG), increasing the size of the larger fragment resulting from the digest by 16 bp. Relative mobility of *Bst*EII-digested bacteriophage λ DNA (sizes in bp) is indicated to the left. **(C)** DNA and deduced amino acid sequence from the edited locus, with deduced amino centered below codons.

The general location of the reductase domain in Lps3 was identified by BLAST analyses, and the active site tyrosine was located in a threonine-tyrosine-lysine triad as described for other reductase domains [16–18] (S2 Fig, S3 Fig). The active site of the reductase domain of Lps3 was mutated via a CRISPR/Cas9-based approach. An sgRNA was designed to target Cas9 to cut at a point two nucleotides from the intended single-nucleotide edit (Fig 2A), and a donor DNA was designed to facilitate mutation of an A residue (part of a TAC Tyr codon) to a T (as part of a TTC Phe codon). The sgRNA was prepared as follows: oligonucleotide 5’-TTCTAATACGACTCACTATA**GACAAACTTGGTCTGGCTGT**GTTTTAGAGCTAGA-3’ was purchased (Eurofins Genomics; Louisville, KY) and used as template in the EnGen sgRNA synthesis kit (New England, BioLabs, Ipswich, MA). The 20-nt target sequence is shown in bold face and is complementary to the coding sequence of that particular region of *lpsC*. In a blastn search of *M. brunneum* genomic sequence data, no off-target site matched the 20-nt sequence of the sgRNA; the closest match returned shared identity at only 14 consecutive nucleotides. The donor DNA was a 96-residue single strand oligonucleotide of the following sequence 5’[phosphate]GAGGAACAAGTGGCGATGCAGTTTGCTGCAGGGGGCATCC**CCT**tCAGCCAGACCAAGTTTGTCGCCGAGTCTCTTGTCAGGCGCGCGGCAGCACGC-3’ also purchased from Eurofins Genomics. The donor DNA was sense orientation relative to *lpsC* and was phosphorylated on the 5’ end. The position of the PAM site (in reverse complement) relative to the co-introduced sgRNA is shown in bold face and the lowercase t represents the nucleotide changed (from the A found in the wild-type locus) upon completion of the mutation process. In a blastn search of *M. brunneum* genomic sequence data, no locus matched the 96-nt sequence of the donor DNA, and the closest off-target match returned in this search shared identity at only 16 consecutive nucleotides.

The donor DNA, EnGen Spy Cas9 NLS (New England BioLabs) complexed with the sgRNA, and a PCR fragment containing a phosphinothricin resistance gene [20] were introduced into spheroplasts of *M. brunneum* ARSEF 9354 according to a transformation method described previously [15,20]. Phosphinothricin-resistant transformants were screened for the desired edit by a polymerase chain reaction-restriction fragment length polymorphism (PCR-RFLP) assay. Template DNA was primed with oligonucleotides 5’-GAAGGAACCAACGTCCGGTCG-3’ and 5’-GTTCATCGGCATTGCACACACC-3’ in a 20 μL PCR with Phusion Green Mastermix (Thermo Scientific, Waltham, MA) and the following program: an initial denaturation at 98°C for 150 s was followed by 35 cycles of template denaturation at 98°C (15 s), primer annealing at 69°C (15 s), and polymerase extension at 72°C (70 s). Two μL of the resulting 355-bp PCR product was then digested with 10 units of the restriction enzyme *Sfc*I (New England BioLabs) for 1 hr in a 10 μL reaction at 37ᵒ C and then analyzed by agarose gel electrophoresis. In separate assays, PCR products were cleaned with the Zymo Clean and Concentrate kit (Zymogen; Irvine, CA) and then sequenced by Sanger technology at Eurofins Genomics.

All genetically modified strains were cultured through at least two rounds of single conidial cultures to promote nuclear homogeneity. Most conidia of *M. brunneum* contain only a single nucleus. Since the fungus is haploid, we only needed to edit one locus per genome.

CRISPR/Cas9-based gene knockouts in late ergot alkaloid pathway genes *easO* and *easP* were made in a strain of *M. brunneum* containing a version of *lpsC* edited as described immediately above. The *easO* knockouts were prepared as described by Steen et al. [15]. Briefly, the *easO* locus was targeted with an sgRNA prepared from oligonucleotide 5’-TTCTAATACGACTCACTATAG**AGTAGTCAAGCACTTTAACT**GTTTTAGAGCTAGA-3’ and the EnGen sgRNA synthesis kit (New England BioLabs). Transformants were selected for hygromycin resistance [20] and screened by PCR with primers 5’-GCCAAACCCTTCGTGGTCG-3’ and 5’-GCAATCAATCGTCGTCCCATCAC-3’ in PCRs as described above (for *lpsC* mutation) but with a primer annealing temperature of 66ᵒ C and a polymerase extension time of 240 seconds. PCR products that differed in length from the corresponding fragment from the wild type were cleaned and Sanger sequenced as described above. Since the fungus is haploid, we only needed to edit one locus per genome.

Strains with a knockout of *easP* were prepared in the *lpsC*-edited background also by following previously published methodology [19]. The sgRNA was prepared as described above but from the following template: 5′-TTCTAATACGACTCACTATAG**TCTGCTCCATGGAGGCTCCT**GTTTTAGAGCTAGA-3′. Potential knockouts were screened by PCR primed with oligonucleotides 5′-CACACTCTACTCCCTCACAAGG-3′ and 5′-CCGCTCCAGGCATCGTCAC-3′. PCR conditions were as described above but with a primer annealing temperature of 66ᵒ C and a polymerase extension time of 80 seconds. PCR products differing in length from those generated from wild type template DNA were cleaned and Sanger sequenced as described above.

### Analysis of ergot alkaloids accumulating in genetically modified strains

To maximize accumulation of ergot alkaloids, conidia from strains of *M. brunneum* were injected into larvae of the model insect *Galleria mellonella* and extracted by bead beating in methanol essentially as previously described [6,15,21]. In this series of experiments, 20 μL of a 3,000 conidia per μL suspension was prepared in modified phosphate-buffered saline solution [21] and injected into the hind-most proleg of larvae of *G. mellonella*. Larva died within four days and ergot alkaloids were extracted on day 4 following inoculation. Ergot alkaloids were analyzed by high-performance liquid chromatograph (HPLC) with fluorescence detection according to methods we had developed and used routinely [6,15,19–23]. Briefly, the solid phase was a C18 silica (Prodigy ODS3; Phenomenex; Torrance, CA) reverse phase column (5-μm particle size; 150 mm x 4.6 mm inside diameter) and the mobile phase was a multilinear binary gradient from 5% acetonitrile + 95% 50 mM aqueous ammonium acetate to 75% acetonitrile + 25% 50 mM aqueous ammonium acetate. Lysergic acid and lysergic acid amides were detected by fluorescence with excitation at 310 nm and emission recorded at 410 nm. Peak identities were defined by retention times relative to peaks in reference strains that had been previously defined by additional instrumental analyses [6,15,19–23]. Identities of major peaks in the edited strains of *M. brunneum* as lysergic acid and lysergyl-alanine were confirmed by high resolution LC-MS according to published methods [6,12] (S4 Fig, S5 Fig). Peak areas were quantified relative to an external standard curve prepared from commercially purchased ergonovine (Sigma-Aldrich; St. Louis, MO), which shares the same fluorophore with lysergic acid and the other lysergic acid derivatives. As a result, data are presented as ‘relative to ergonovine’ as opposed to being absolute quantities. Accumulation of individual alkaloids was calculated on a molar basis and expressed as a percent of the summed total ergot alkaloids. Because inter-strain variances differed significantly in Brown-Forsythe tests, differences in means were analyzed nonparametrically by Wilcoxon rank sum tests followed by Steel-Dwass nonparametric multiple comparison tests. Statistical analyses were performed with JMP version 18 (SAS; Cary, NC).

We used an H_2_^18^O assay to investigate whether the lysergic acid observed accumulating in the edited mutant of *M. brunneum* was on-pathway intermediate (in which case both oxygens would be derived from molecular oxygen) or whether it had been hydrolyzed from the Lps complex or more complex lysergic acid derivatives (in which case one oxygen would be derived from water). In the presence of H_2_^18^O, lysergic acid that had been hydrolyzed from a larger molecule would produce a molecular ion of [M + H]^+^ = 271 as opposed to [M + H]^+^ = 269 for lysergic acid as an on-pathway intermediate. The edited strain of *M. brunneum* (strain e24) or an *lpsB* knockout strain [20], which served as a negative control for its inability to bind lysergic acid or incorporate it into more complex lysergic acid derivatives, were injected into larvae of *G. mellonella*. Half of the larvae, which have a volume of approximately 200 µL, were subsequently injected on both two days and three days after inoculation with 40 µL H_2_^18^O (97% ^18^O, Sigma part number 329878), and the other half received 40 µL of plain distilled water on those days. On day 4, ergot alkaloids were extracted from larvae by bead beating, and extracts were analyzed by LC/MS on a Thermo LCQ Deca XP plus mass spectrometer as previously described [15]. Abundance of ions at [M + H]^+^ = 269 and [M + H]^+^ = 271 were recorded in the lysergic acid peak, and the percent of [M + H]^+^ = 271 relative to [M + H]^+^ = 269 was calculated. In the extracts of the edited strain *M. brunneum* e24, we also compared the relative abundances of ions at [M + H]^+^ = 273, representing dimethylallyltryptophan (DMAT), the first intermediate of the ergot alkaloid pathway, and [M + H]^+^ = 275, corresponding to ^18^O-containing DMAT molecules. Data were checked for unequal variance with a Brown-Forsythe test and then analyzed by one-way ANOVA with JMP software (SAS, Cary, NC).

## Results

### Editing of the reductase domain of Lps3

We used a CRISPR/Cas9-based approach to edit a single nucleotide in the reductase domain-encoding-region of the gene *lpsC* encoding Lps3 (Fig 2). An edited mutant was identified in an initial screen of transformed colonies by a PCR-RFLP strategy. The screen was made possible because the planned editing event eliminated an *Sfc*I recognition site (CTRYAG) and increased the length an *Sfc*I fragment by 16 bp (Fig 2AB). Sanger sequencing of the mutated locus demonstrated that the A residue of the original TAC (Tyr) codon had been mutated to T, resulting in a TTC (Phe) codon (Fig 2C). Subsequent Sanger sequencing from multiple primers across the entire *lpsC* gene demonstrated that the A to T mutation––resulting in a Y1621F mutation in the protein––was the only mutation in the allele of *lpsC* contained in the edited mutant strain *M. brunneum* e24 (GenBank accession PP064004) compared to the allele in the wild type, *M. brunneum* strain ARSEF 9354 (GenBank accession OP261544).

### Biochemical consequences of editing Lps3 reductase domain Tyr to Phe

**Fig 3:**
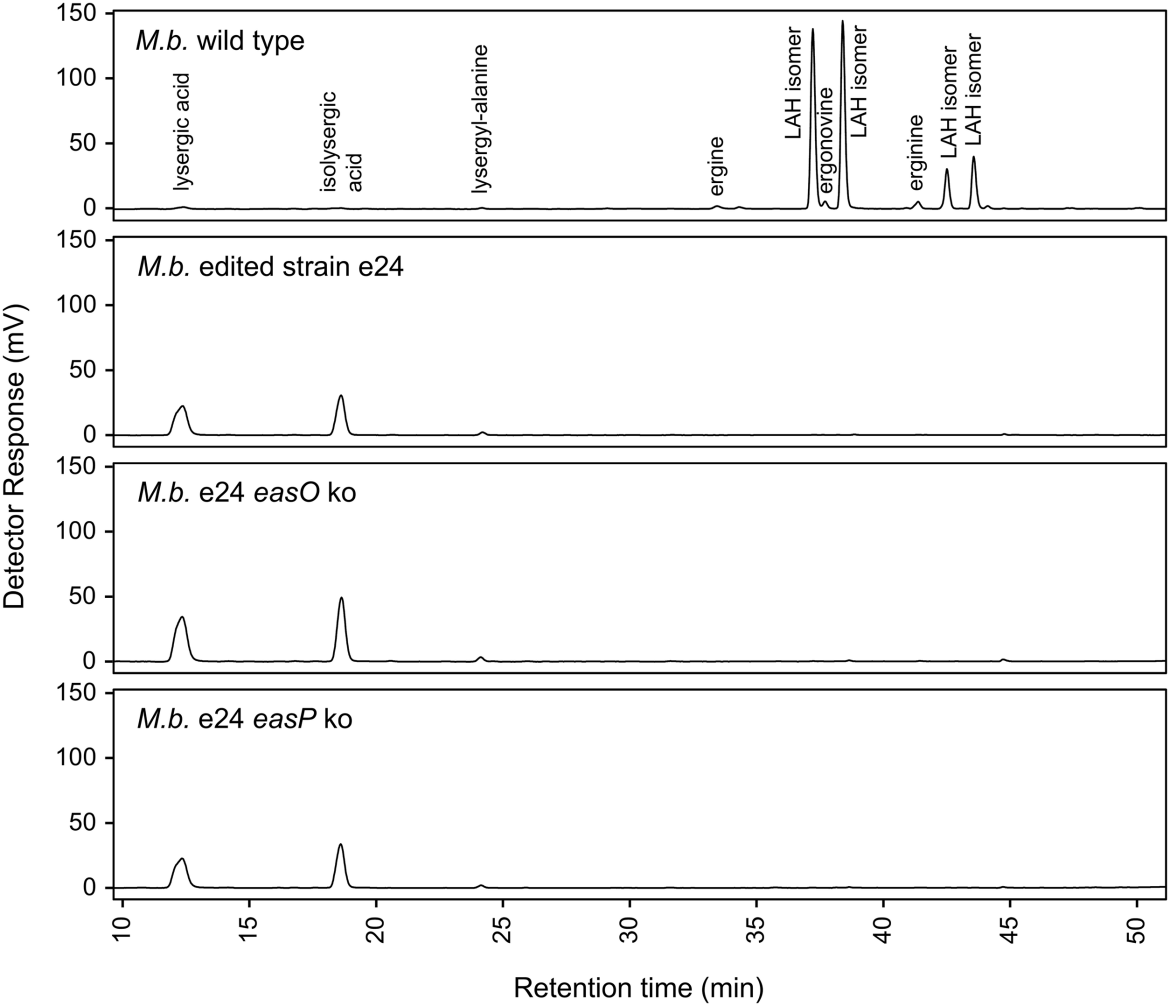
HPLC of wild-type and modified strains of *M. brunneum.* Most lysergic acid derivatives stereoisomers in protic solvents, and stereoisomers are labeled here (including erginine as the stereoisomer of ergine).

**Fig 4:**
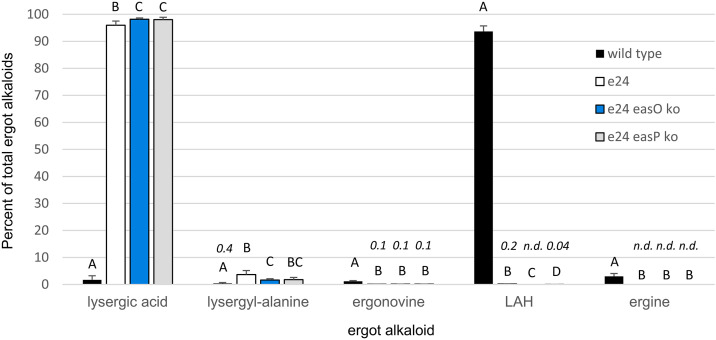
Accumulation of lysergic acid and lysergic acid derivatives in larvae of *G. mellonella* infected with the *M. brunneum* ARSEF 9354 (wild type), edited strain *M. brunneum* strain e24 in which the reductase domain of Lps3 carries the Y1621F mutation, and an *easO* or an *easP* knockout in the *M. brunneum* edited strain e24 background. Ergot alkaloids are expressed as mean molar percentage of the total summed ergot alkaloid concentration in a given strain and relative to an external standard curve of ergonovine. Error bars represent standard deviation. Capital letters reflect differences in means (within each alkaloid type) as calculated in Steel-Dwass non-parametric multiple comparison tests. Values for means less than 1% of total ergot alkaloids are presented in italicized numerals; n.d., not detected.

To maximize ergot alkaloid accumulation, spores from the wild-type strain *M. brunneum* ARSEF 9354 and its edited derivative *M. brunneum* strain e24 bearing the Y1621F mutation (altering the active site Tyr of the reductase domain in Lps3 to Phe) were injected into larvae of the insect *G. mellonella*. The ergot alkaloid profile of the edited strain differed significantly from that of the wild type (Figs 3 and 4). Edited strain *M. brunneum* e24 accumulated only trace quantities of LAH––the pathway end product and most abundant ergot alkaloid in *M. brunneum* ARSEF 9354––and instead accumulated primarily the pathway intermediate lysergic acid (and its stereoisomer, which forms in protic solvents [22]) (Figs 3 and 4). The near elimination of LAH in the edited mutant shows that the reductase domain has a significant role in LAH biosynthesis. Potential reasons for the presence of residual trace levels of LAH and ergonovine are addressed in studies described in a later section. The lack of detectable ergine follows logically from the extremely low levels of LAH. Ergine forms as a spontaneous hydrolysis product of LAH, and if LAH levels are borderline detectable then ergine will not be observed.

### Biochemical consequences of knocking out other late pathway genes in the *M. brunneum* strain e24 (Y1624F) background

To investigate how the reductase domain of Lps3 works relative to other late pathway enzymes EasO (encoding a Bayer-Villiger monooxygenase required for LAH accumulation [15,24]) and EasP (encoding an alpha/beta hydrolase fold protein that quantitatively affects LAH accumulation [19]), we made knockouts of *easO* and *easP* separately in the *M. brunneum* strain e24 (Y1621F) genetic background (S6 Fig, S7 Fig).

The ergot alkaloid profile of the *easO* knockout in the edited *M. brunneum* strain e24 background was dominated by lysergic acid (over 95% of the observed ergot alkaloid content), with lysergyl-alanine (~2% of total ergot alkaloids) as the only other ergot alkaloid accumulating to over 1% of the total measured ergot alkaloids. The lack of detectable LAH in *easO* knockout was consistent with previous data [15] and the proposed role of an *easO*-encoded Bayer-Villiger monooxygenase in LAH biosynthesis [4,15,24]. The trace levels of ergonovine remaining were similar to those observed in the *M. brunneum* e24 background described above, indicating some residual reductase activity in the edited Y1621F background strain.

The ergot alkaloid profile of *easP* knockouts in the edited strain e24 background also were dominated by lysergic acid. The presence of trace residual LAH in *easP* ko mutant suggests that residual LAH in the edited parent strain (*M. brunneum* strain e24) is due to factors other than, or in addition to, the potential hydrolysis of LAH from enzyme-bound precursor by EasP. The already low level of LAH in the reductase domain-edited strain e24 were significantly decreased by the knockout of *easP* supporting results from a previous study [19]. Low residual levels of ergonovine were as described above for the edited strain *M. brunneum* e24.

The trace level of residual LAH in the edited Y1621F mutant *M. brunneum* e24 and its *easP*-knockout derivative could result from the presence of wild-type nuclei in these multinucleate fungi or from some other activity not accounted for in these experiments. Several observations do not support the presence of wild-type nuclei in *M. brunneum* strain e24. Two subsequent rounds of culturing from single conidia did not eliminate the residual LAH. Visual analysis of chromatograms from Sanger sequencing of the locus after passaging through insects did not reveal the presence of contaminating wild type nuclei (S8 Fig). The complete lack of detectable LAH in the *easO* knockout indicates a lack of wild-type nuclei in that particular strain (Fig 4), whereas trace residual ergonovine in that very same strain indicates unexplained residual reductase activity.

### Investigation of lysergic acid as an intermediate compared to lysergic acid derived via hydrolysis from more complex sources

**Fig 5:**
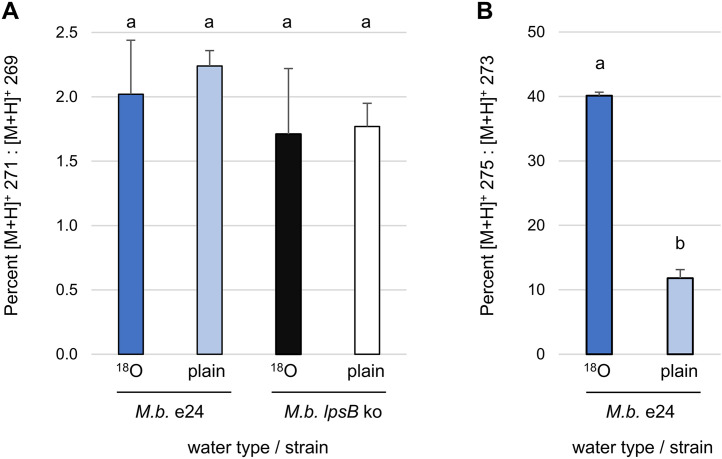
Relative abundance of heavy ions ([M + H]^+^ + 2) relative to molecular ions in samples of *M. brunneum* strains fed H_2_^18^O compared to plain water. **(A)** Abundance of [M + H]^+^ = 271 ion (corresponding to molecular ion of lysergic acid + 2 Daltons) relative to [M + H]^+^ = 269 ion (corresponding to molecular ion of lysergic acid) in peak corresponding to lysergic acid. **(B)** Abundance of [M + H]^+^ = 275 ion (corresponding to molecular ion of dimethylallylpyrophosphate [DMAT] + 2 Daltons) relative to [M + H]^+^ = 273 ion (corresponding to molecular ion of DMAT) in peak corresponding to DMAT. Error bars represent standard deviation. Lower case letters reflect differences in means (within each alkaloid type) as calculated by ANOVA.

Results of an H_2_^18^O-feeding experiment suggested that the lysergic acid observed accumulating in the Y1621F edited strain, *M. brunneum* strain e24, was intermediate from the ergot alkaloid biosynthesis pathway (with both of its oxygens derived from O_2_) and had not been hydrolyzed from the Lps complex or some other lysergic acid derivative in which case one of its oxygens would have been derived from water. The potential presence of water-derived oxygen in lysergic acid was estimated by comparing the abundance of [M + H]^+^ = 271 (corresponding to the molecular ion of lysergic acid plus two Daltons to account for ^18^O) relative to the abundance of [M + H]^+^ = 269, the molecular ion for lysergic acid. In *M. brunneum* strain e24, there was no significant difference in the abundance of [M + H]^+^ = 271 ion relative to [M + H]^+^ = 269 in the peak corresponding to lysergic acid in samples fed ^18^O water relative to plain water (*P* = 0.42) (Fig 5A). An *lpsB* knockout strain of *M. brunneum*, in which the coding sequences for the lysergic acid-activating enzyme Lps2 were disrupted prior to the adenylation domain [20], served as a negative control because it cannot bind lysergic acid to Lps2 or incorporate it into other derivatives. In this strain the percentage of [M + H]^+^ = 271 relative to [M + H]^+^ = 269 was similar in samples injected with plain versus ^18^O water (*P* = 0.85) and also similar to the percentage of [M + H]^+^ = 271 to [M + H]^+^ = 269 observed in strain e24 (*P* = 0.46) (Fig 5A). The lack of detectable water-derived oxygen in the lysergic acid pool accumulating in *M. brunneum* strain e24 suggests that the observed lysergic acid had not been hydrolyzed from the Lps enzyme complex or other lysergic acid derivatives. Analysis of the relative abundance of the molecular ion for the ergot alkaloid pathway intermediate dimethylallyltryptophan (DMAT; [M + H]^+^ = 273) relative to a predicted heavy oxygen version of DMAT ([M + H]^+^ = 275) provided evidence of detectable, though potentially indirect, incorporation of ^18^O into a fungal metabolite in these same fungus-infected larvae (Fig 5B). Treatments injected with H_2_^18^O contained a significantly higher ratio of [M + H]^+^ = 275 relative to [M + H]^+^ = 273 than controls injected with typical distilled water (*P* < 0.0001).

## Discussion

Our results provide strong evidence that the reductase domain of Lps3 in *M. brunneum* plays a significant role in the synthesis of LAH. Near elimination of LAH accumulation was observed in response to a single amino acid change at the proposed active site of the reductase domain. The accumulation of the pathway intermediate lysergic acid, as opposed to later pathway intermediates, suggests that without a functioning reductase domain the Lps complex (comprised of lysergic acid-activating enzyme Lps2 and multifunctional Lps3) does not bind substrate for subsequent rounds of catalysis. Such a phenotype might arise if substrates from a preceding cycle were still bound to the enzyme.

Our proposed model for the final stages of LAH biosynthesis, starting from lysergyl-alanine bound to the Lps complex (Fig 1), involves the Bayer-Villiger monooxygenase EasO and the reductase domain of Lps3 [4,15]. Genetic and biochemical data indicate that the Bayer-Villiger monooxygenase encoded by *easO* inserts an oxygen between the alpha carbon and the carbonyl carbon of the alanine portion of Lps3-bound lysergyl-alanine [4,15,24]. We propose that the reductase domain of Lps3 then reduces the carbonyl carbon of that diester intermediate (just as it does during ergonovine synthesis [13]) and releases either of two products: 1) LAH if reduction breaks the bond between the inserted oxygen and the carbonyl carbon; or, 2) a formate ester of LAH if reduction breaks the bond between the carbonyl carbon and the sulfur of the 4’-phosphopantetheine cofactor (Fig 1). In the former case, a formyl group would remain attached to the sulfur group of the 4’-phosphopantetheine cofactor and need to be removed to allow another round of catalysis. In the latter case, a formyl group would need to be removed from the LAH formate ester to produce LAH. One potential candidate for either of these reactions is the alpha/beta hydrolase fold protein encoded by *easP*, which has been shown to increase yield of LAH while not being essential for LAH synthesis [19]. The hypothesized formate ester of LAH has never been directly observed; strains with *easP* knockouts did, however, have significantly increased concentrations of ergine (the simple amide of lysergic acid) which could result from hydrolysis of the presumably labile LAH formate ester [19]. Another possible function for EasP is to directly hydrolyze LAH from the diester intermediate formed by EasO activity (Fig 1). This proposed function would be an alternative to the process of reduction of LAH from the diester intermediate by the Lps3 reductase domain and thus would not necessarily increase yield of LAH. Functional analyses of *easP* by gene knockouts (reference [19] and the present study) have shown that EasP increases yield of LAH but have not provided evidence of a mechanism.

Abundant accumulation of the Lps complex substrate lysergic acid in the edited strain and both of its knockout derivatives suggests that the Y1621F mutation is severely debilitating to the Lps complex. One potential interpretation consistent with these data is that the reductase domain liberates LAH (similar to the way it does ergonovine [13]) from intermediate bound to the 4’-phosphopantetheine cofactor; thus in the absence of functioning reductase domain substrates remain bound to the enzyme, preventing subsequent rounds of catalysis. An extension of this hypothesis is that the reductase domain also may be responsible for reducing (and thus removing) other residues bound to the sulfur molecule of the 4’-phosphopantetheine cofactor. In other words, the reductase domain may reduce the carbonyl carbon attached to the sulfur group of the 4’-phosphopantetheine cofactor without regard for the remainder of the attached substrate. For example, release of LAH by reduction would leave a formyl group attached to the sulfur molecule of the 4’-phosphopantetheine cofactor (Fig 1). This formyl group would need to be removed, potentially via reduction, to restore the sulfhydryl group to prepare Lps3 to accept more substrate for another round of catalysis. In some other nonribosomal peptide synthetase systems, a type II thioesterase (TEII) removes failed reaction intermediates from the sulfur group of the group of the 4’-phosphopantetheine cofactor [25]. A search for proteins encoded in the *M. brunneum* genome similar to fungal TEIIs such as LovG of *Aspergillus terreus* (GenBank accessions Q0C8M2) [26], GenBank accession QCC62998 associated with BII-rafflesfungin from *Phoma* species [27], and GenBank accession KPA42378 from *Fusarium langsethiae* [28] yielded matches to deduced proteins with 29–31% sequence identity––a level not providing compelling evidence for functional equivalence among the large family of alpha/beta hydrolase fold proteins. Moreover, in H_2_^18^O feeding experiments we did not detect a significant fraction of the lysergic acid that accumulated in the *M. brunneum* e24 mutant as derived from hydrolysis from the Lps complex (or other lysergic acid derivatives). This observation suggests that lysergic acid had not been repeatedly bound and hydrolyzed from Lps2 and is consistent with a lack of an available type II thioesterase to liberate enzyme-bound lysergic acid from a stalled Lps2. Analysis of ions related to the ergot alkaloid pathway intermediate DMAT in these same extracts provided evidence of availability of H_2_^18^O to the fungus but must be considered in light of the limitations of DMAT as a positive control. The oxygens of DMAT are derived from the carboxylic acid of tryptophan. Although insects do not synthesize tryptophan, the oxygens of tryptophan are derived from serine which would be among the metabolite pool of both the fungus and insect. Thus, the ^18^O detected in DMAT could have been incorporated directly from fungal metabolism of H_2_^18^O or indirectly through fungal acquisition of ^18^O-labeled serine from the insect hemocoel.

The small amount of LAH in *M. brunneum* strain e24 and the strain e24 *easP* knockout, as well as the trace amounts of ergonovine in all the *M. brunneum* strain e24 derivatives, indicate some residual activity capable of reducing product from enzyme-bound lysergyl-alanine derivatives. Attempts to remove any contaminating wild-type nuclei (one possible source of such activity) from modified strains did not decrease the amount of residual ergonovine or LAH, and sequence analysis of the edited site in the various *M. brunneum* strain e24 derivatives provided no evidence of contaminating wild-type nuclei. We have no further basis for speculating on the source of residual reductase activity.

We chose *M. brunneum* for this study because it is a genetically manipulable producer of LAH. Many other researchers study *Metarhizium* species because of their roles as rhizosphere-associated biocontrol agents of insects [29,30]. Several studies have used CRISPR/Cas9-based approaches to knock genes out in *Metarhizium* species for functional analyses [15,19,20,31–34]; however, to our knowledge, our study is the first to employ a CRISPR/Cas9-based gene editing approach to modify a single amino acid in a *Metarhizium* species. In our analyses, we screened 46 transformants by PCR-RFLP and found two transformants that had patterns consistent with the intended single-nucleotide mutation. In both cases Sanger sequencing confirmed the single nucleotide substitution encoded in the donor DNA. One of the mutants was *M. brunneum* strain e24, which was studied herein; the second edited strain had an ergot alkaloid profile like that of *M. brunneum* strain e24, but we did not sequence the entire *lpsC* gene for off target mutations or develop *easO* or *easP* mutations in that second edited strain. We made no attempt to optimize our editing system and suspect the efficiency of nucleotide editing in *Metarhizium* species can be improved with further experimentation.

## Conclusions

The editing approach used in this study provides strong evidence for an important role in LAH biosynthesis for the tyrosine residue at the proposed active site of the reductase domain of Lps3. The data thus support a key functional role for the reductase domain of Lps3 in LAH synthesis. Moreover, the results suggest that without a functional reductase domain, the multifunctional Lps complex does not bind substrate for additional rounds of catalysis. Additional biochemical approaches will be necessary to further clarify the final steps of lysergic acid amide synthesis. The study also demonstrates applicability of a general strategy for editing individual nucleotides in a *Metarhizium* species via a CRISPR/Cas9 approach.

## Supporting information

S1 Raw images Original gels supporting Fig 2B, S6 Fig, and S7 Fig. (PDF)

S2 Fig Functional domains of Lps3 indicated with highlighted text. Yellow-highlighted region corresponds to the adenylation domain. Purple highlight represents thiolation domain. Green region corresponds to the condensation domain. The thioester reductase domain is highlighted blue, and its deduced threonine-tyrosine-lysine catalytic triad is highlighted yellow against the blue background (as defined with BLASTp alignment with conserved thioester reductase domain protein family model TIGR01746; S3 Fig).
(TIF)

S3 Fig Alignment of the reductase domain of Lps3 of *M. brunneum* ARSEF 9354 (from GenBank accession OP261544) with conserved thioester reductase domain protein family model TIGR01746. Identical amino acids occupying conserved positions are shown in red. The threonine-tyrosine-lysine catalytic triad is highlighted yellow.
(TIF)

S4 Fig Accumulation of an analyte consistent with the molecular ion and fragments associated with lysergic acid in larvae of *G. mellonella* infected with the *M. brunneum* strain e24 (Y1621F). Analyte was detected by high-resolution liquid chromatography-mass spectrometry (LC-MS) [6,12]. Observed mass of molecular ion (269.1277–1.007 for mass of ionizing proton) relative to theoretical mass of lysergic acid (268.1212) yield a mass error of −1.9 ppm.
(TIF)

S5 Fig Accumulation of an analyte consistent with the molecular ion and fragments associated with lysergyl-alanine in larvae of *G. mellonella* infected with the *M. brunneum* strain e24 (Y1621F). Analyte was detected by LC-MS [6,12]. Observed mass of molecular ion (340.1652–1.007 for mass of ionizing proton) relative to theoretical mass of lysergyl-alanine (339.1583) yield a mass error of −0.3 ppm.
(TIF)

S6 Fig CRISPR/Cas9-induced knockout mutation in *easO* of *M. brunneum* edited strain e24. On left of panel are PCR products from genomic DNA of *M. brunneum* strain e24 (e24) and *easO* knockout (ko) primed with oligonucleotides 5’-GCCAAACCCTTCGTGGTCG-3’ and 5’-GCAATCAATCGTCGTCCCATCAC-3’. Relative mobility of relevant fragments of *Bst*EII-digested bacteriophage λ (sizes in kb) are indicated. The right of the panel shows DNA sequence of *easO* after CRISPR/Cas9 mutagenesis, with sequences of the hygromycin resistance-conferring plasmid pBChygro (incorporated into the locus during repair) highlighted blue. sgRNA target sequence is underlined and PAM site is shown in bold face. The abbreviation [nnnnnn] represents 2000 nt of the inserted construct that were omitted to simplify the presentation.
(TIF)

S7 Fig CRISPR/Cas9-induced knockout mutation in *easP* of *M. brunneum* edited strain e24. On left of panel are PCR products from genomic DNA of *M. brunneum* strain e24 (e24) and an *easP* knockout (ko) primed with oligonucleotides 5’-CACACTCTACTCCCTCACAAGG-3’ and 5’-CCGCTCCAGGCATCGTCAC-3’. Relative mobility of relevant fragments of *Bst*EII-digested bacteriophage λ (sizes in kb) are indicated. The right of the panel shows DNA sequence of *easP* after CRISPR/Cas9 mutagenesis, with sequences of the hygromycin resistance-conferring plasmid pBChygro (incorporated into the locus during repair) highlighted yellow. sgRNA target sequence is underlined and PAM site is shown in bold face. The abbreviation [nnnnnn] represents 2000 nt of the inserted construct that were omitted to simplify the presentation.
(TIF)

S8 Fig Chromatograms from Sanger sequencing reactions from the edited region of *lpsC* loci PCR-amplified from DNA prepared from cultures of the indicated strains. The T residue edited from the original A residue is indicated with a red arrow in each chromatogram. No trace of the wild type-encoded A residue is evident in any chromatogram.
(TIF)

S9 Raw data Data from which means and standard deviations graphed in Fig 4 and Fig 5 are derived. Data are supplied in a Microsoft Excel file with separate tabs for Fig 4 and Fig 5.
(XLSX)
