# Supplementary material for: Gene editing of the thioester reductase step in the biosynthesis of lysergic acid amides
Source: PLoS One. 2025 Oct 29;20(10):e0334651. doi: 10.1371/journal.pone.0334651 (PMC12571304; doi:10.1371/journal.pone.0334651)
Supplement: S1 Raw images — (PDF) [file pone.0334651.s001.pdf]

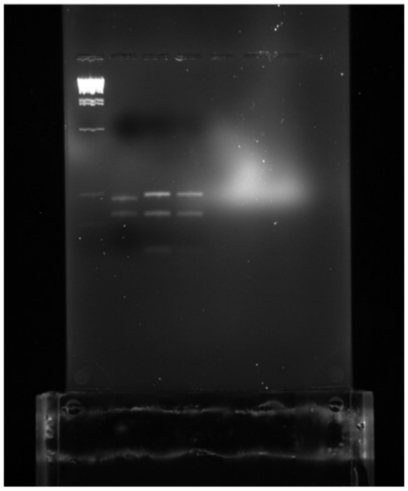

Original gel supporting Fig 2B

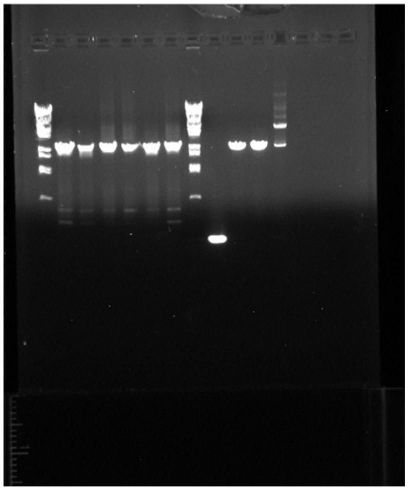

Original gel supporting S6 Fig

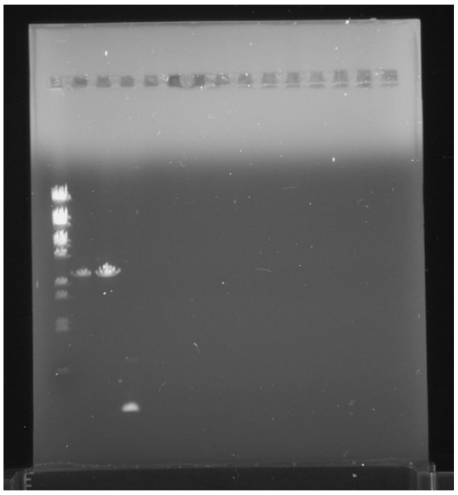

Original gel supporting S7 Fig

All images captured with a Bio-Rad Molecular Imager Gel Doc XR+ with Image Lab Software
