# Supplementary figures and images for: Gene editing of the thioester reductase step in the biosynthesis of lysergic acid amides
Source: PLoS One. 2025 Oct 29;20(10):e0334651. doi: 10.1371/journal.pone.0334651 (PMC12571304; doi:10.1371/journal.pone.0334651)

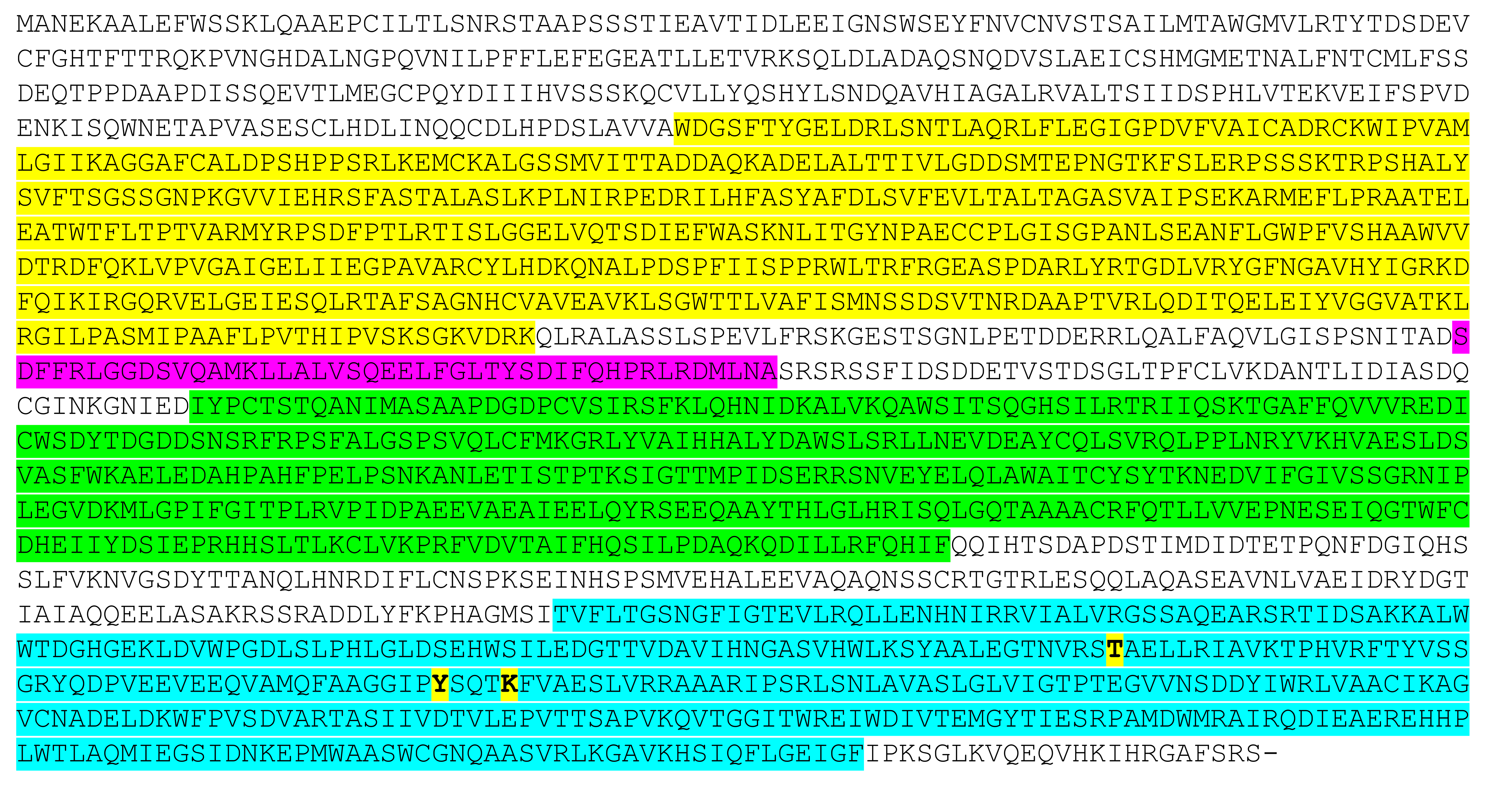

Supplement: S2 Fig — Yellow-highlighted region corresponds to the adenylation domain. Purple highlight represents thiolation domain. Green region corresponds to the condensation domain. The thioester reductase domain is highlighted blue, and its deduced threonine-tyrosine-lysine catalytic triad is highlighted yellow against the blue background (as defined with BLASTp alignment with conserved thioester reductase domain protein family model TIGR01746; S3 Fig). (TIF) [file pone.0334651.s002.tif]

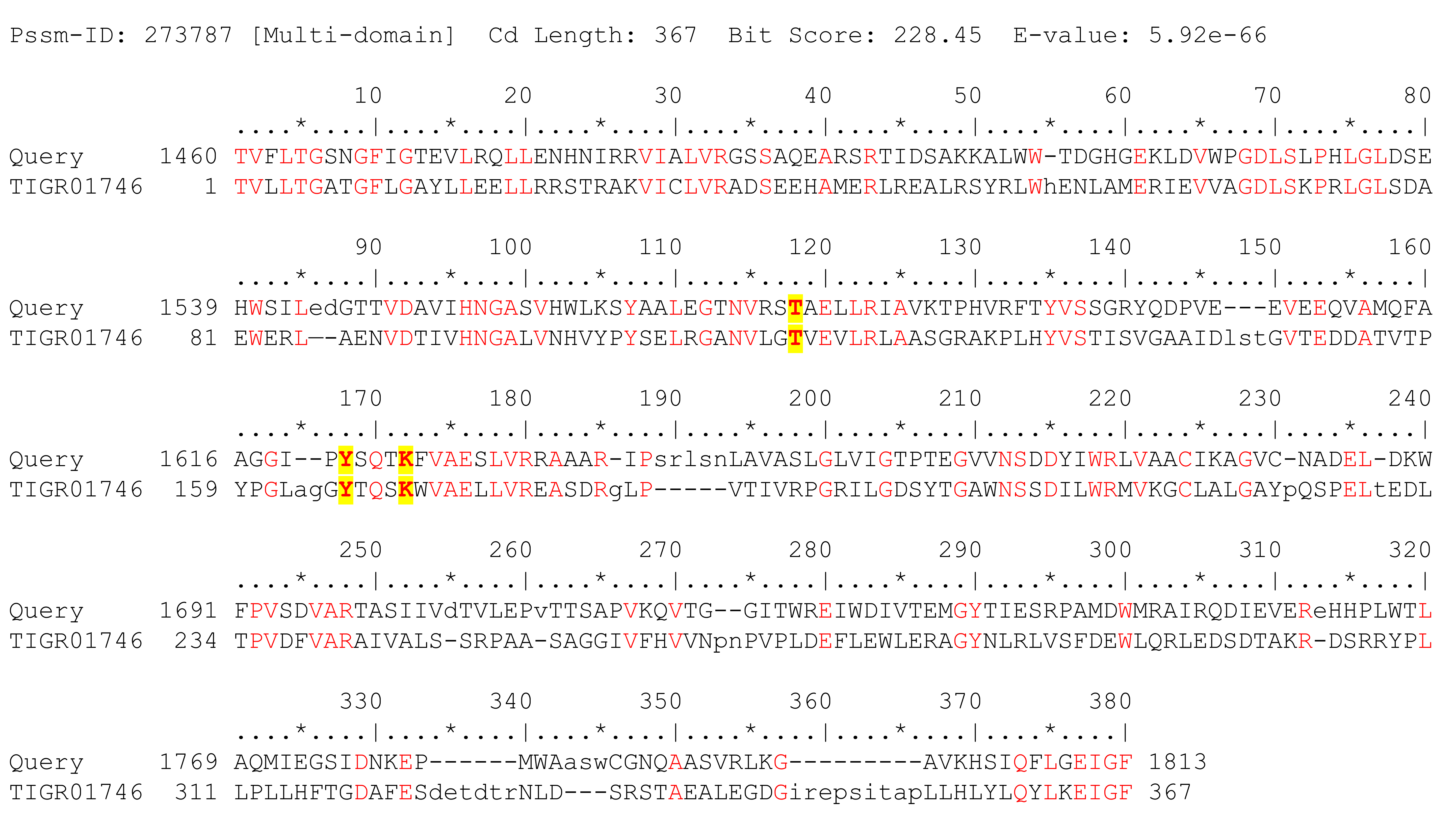

Supplement: S3 Fig — Identical amino acids occupying conserved positions are shown in red. The threonine-tyrosine-lysine catalytic triad is highlighted yellow. (TIF) [file pone.0334651.s003.tif]

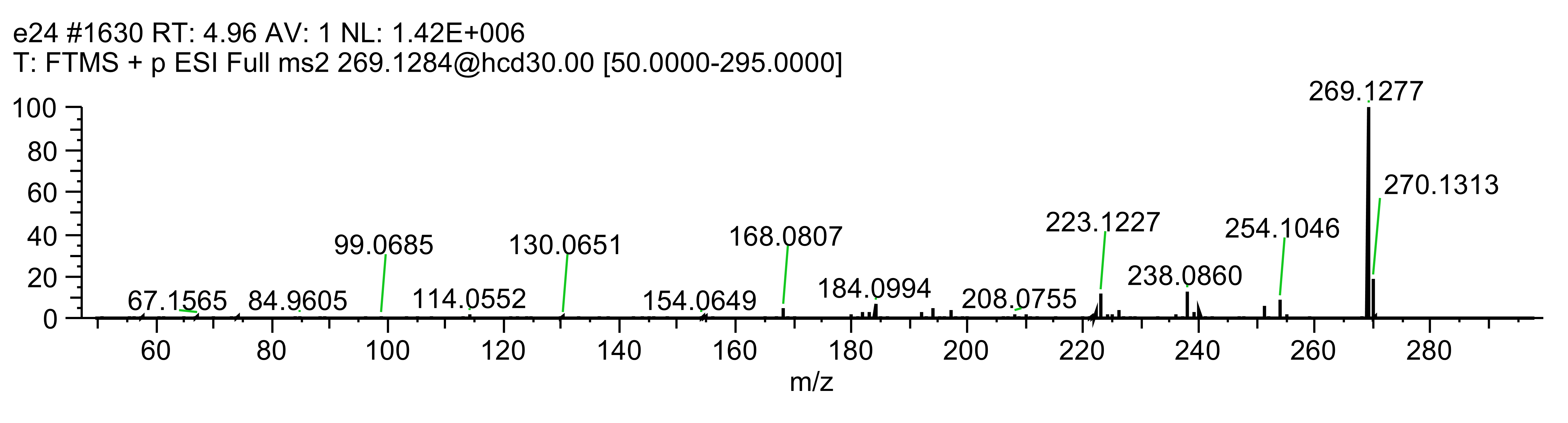

Supplement: S4 Fig — Analyte was detected by high-resolution liquid chromatography-mass spectrometry (LC-MS) [6,12]. Observed mass of molecular ion (269.1277–1.007 for mass of ionizing proton) relative to theoretical mass of lysergic acid (268.1212) yield a mass error of −1.9 ppm. (TIF) [file pone.0334651.s004.tif]

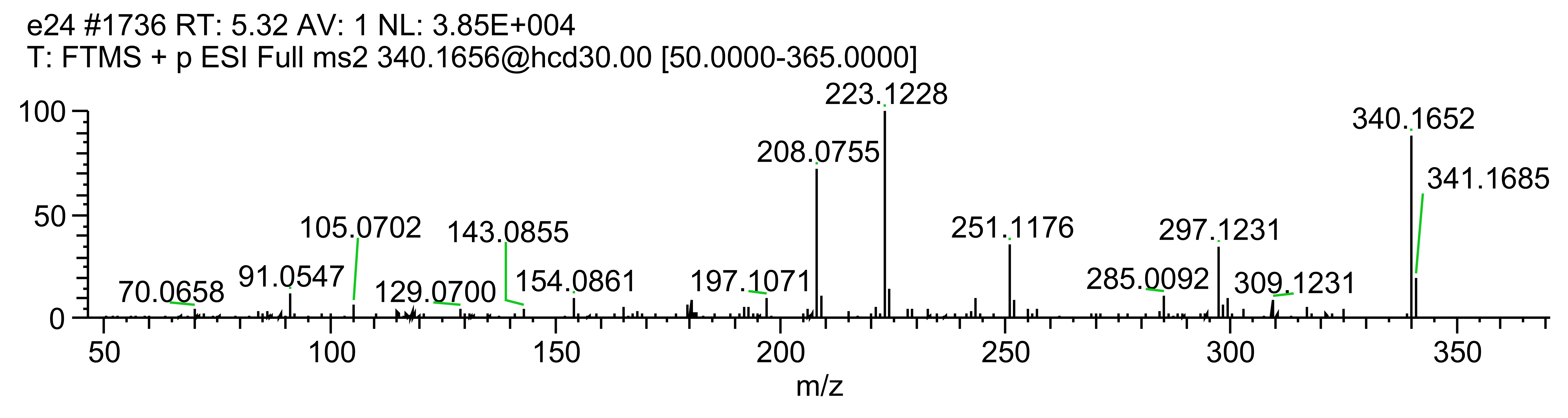

Supplement: S5 Fig — Analyte was detected by LC-MS [6,12]. Observed mass of molecular ion (340.1652–1.007 for mass of ionizing proton) relative to theoretical mass of lysergyl-alanine (339.1583) yield a mass error of −0.3 ppm. (TIF) [file pone.0334651.s005.tif]

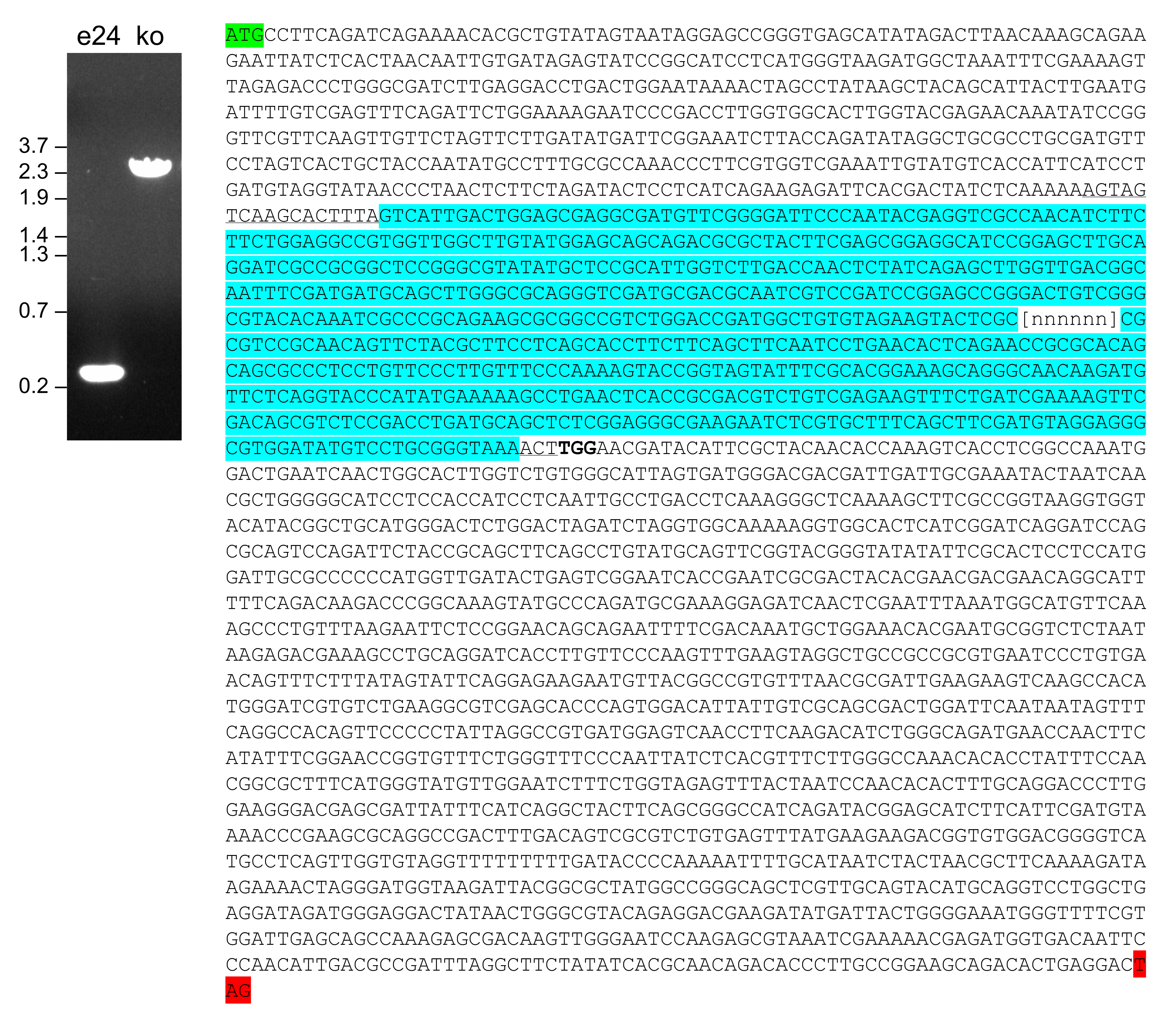

Supplement: S6 Fig — On left of panel are PCR products from genomic DNA of M. brunneum strain e24 (e24) and easO knockout (ko) primed with oligonucleotides 5’-GCCAAACCCTTCGTGGTCG-3’ and 5’-GCAATCAATCGTCGTCCCATCAC-3’. Relative mobility of relevant fragments of BstEII-digested bacteriophage λ (sizes in kb) are indicated. The right of the panel shows DNA sequence of easO after CRISPR/Cas9 mutagenesis, with sequences of the hygromycin resistance-conferring plasmid pBChygro (incorporated into the locus during repair) highlighted blue. sgRNA target sequence is underlined and PAM site is shown in bold face. The abbreviation [nnnnnn] represents 2000 nt of the inserted construct that were omitted to simplify the presentation. (TIF) [file pone.0334651.s006.tif]

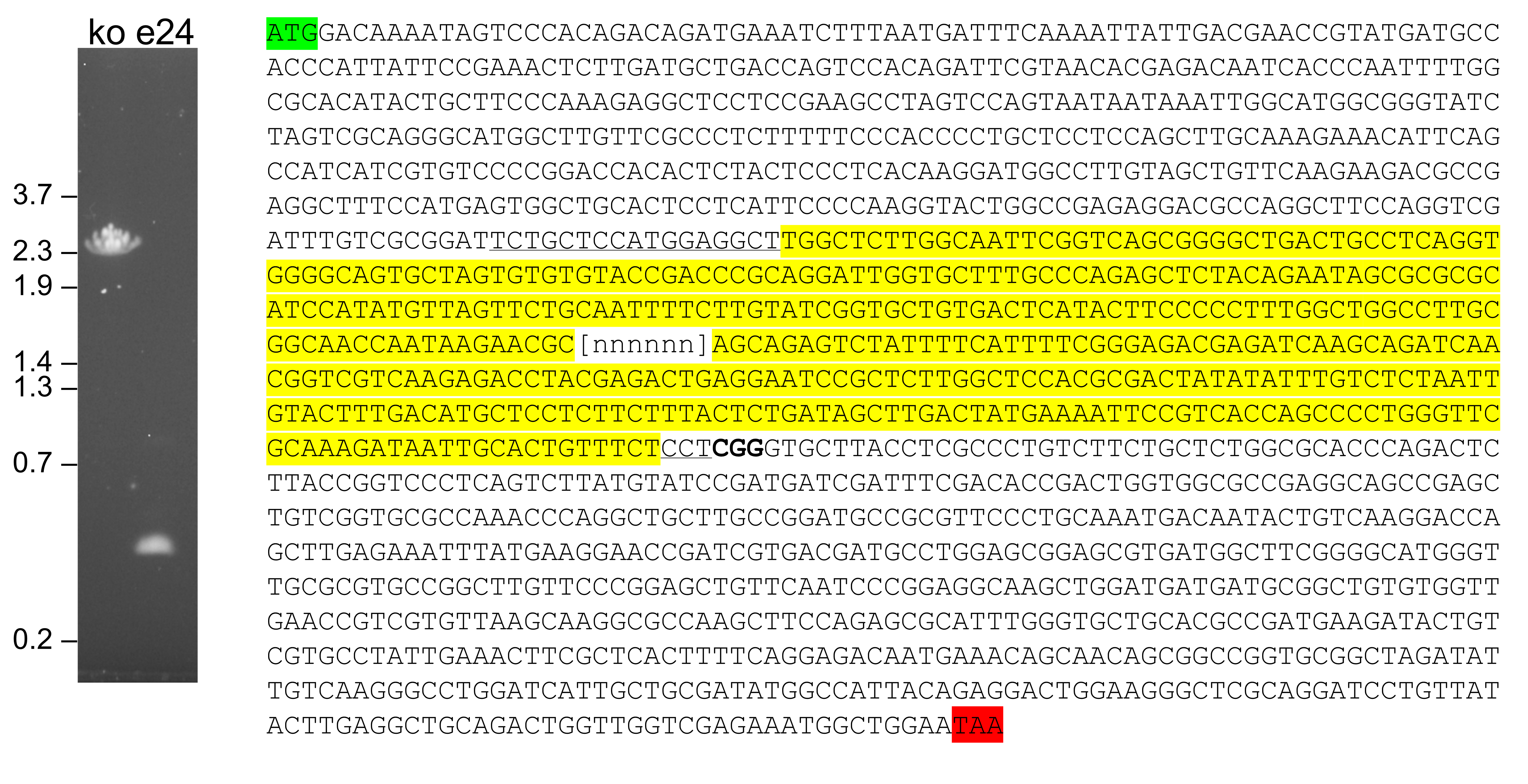

Supplement: S7 Fig — On left of panel are PCR products from genomic DNA of M. brunneum strain e24 (e24) and an easP knockout (ko) primed with oligonucleotides 5’-CACACTCTACTCCCTCACAAGG-3’ and 5’-CCGCTCCAGGCATCGTCAC-3’. Relative mobility of relevant fragments of BstEII-digested bacteriophage λ (sizes in kb) are indicated. The right of the panel shows DNA sequence of easP after CRISPR/Cas9 mutagenesis, with sequences of the hygromycin resistance-conferring plasmid pBChygro (incorporated into the locus during repair) highlighted yellow. sgRNA target sequence is underlined and PAM site is shown in bold face. The abbreviation [nnnnnn] represents 2000 nt of the inserted construct that were omitted to simplify the presentation. (TIF) [file pone.0334651.s007.tif]
